# Brain structural and functional changes during menstrual migraine: Relationships with pain

**DOI:** 10.3389/fnmol.2022.967103

**Published:** 2022-09-14

**Authors:** Zi-wen Wang, Zi-han Yin, Xiao Wang, Yu-tong Zhang, Tao Xu, Jia-rong Du, Yi Wen, Hua-qiang Liao, Yu Zhao, Fan-rong Liang, Ling Zhao

**Affiliations:** ^1^Acupuncture and Tuina School, Chengdu University of Traditional Chinese Medicine, Chengdu, China; ^2^Sichuan Provincial Acupuncture Clinical Medicine Research Center, Chengdu, China; ^3^Hospital of Chengdu University of Traditional Chinese Medicine, Chengdu, China; ^4^Chengdu Integrated Traditional Chinese Medicine and Western Medicine Hospital, Chengdu, China

**Keywords:** anterior cingulum cortex, functional connectivity, menstrual migraine, pain, voxel-based morphometry (VBM)

## Abstract

**Objectives:**

Menstrual migraine (MM) is a special type of migraine associated with the ovarian cycle, which imposes a marked burden on female patients. However, the pathogenesis of MM is not completely understood. We investigated gray matter volume (GMV) and functional connectivity (FC) alterations in patients with MM to explore whether there are changes in resting-state FC (rsFC) in brain regions with structural GMV abnormalities and investigated their relevance to pain and concomitant symptoms.

**Methods:**

Seventy-five patients with MM and 54 female healthy controls underwent functional magnetic resonance imaging and examination. The patients completed a patient’s headache diary, which included the frequency of migraine attacks, a visual analog scale for pain, a self-rating anxiety scale, and a self-rating depression scale. We used voxel-based morphometry (VBM) to examine the GMV differences between the MM and healthy control groups. The identified brain areas were selected as seeds to assess functional changes in the MM group. Correlation analysis between the altered VBM/rsFC and clinical outcomes was performed.

**Results:**

Compared with healthy controls, patients with MM showed decreased GMV in the right anterior cingulum cortex (ACC) and increased GMV in the right superior parietal cortex. Pearson’s correlation analysis illustrated that only GMV in the right ACC was associated with visual analogue scale pain scores in the MM group. RsFC with the ACC as the seed showed that patients with MM exhibited increased FC between the ACC and the left inferior temporal gyrus, bilateral angular gyrus, and right precuneus. Correlation analysis showed that the change in FC between the right ACC and the right precuneus was positively correlated with headache frequency, and the change in FC between the right ACC and the right angular gyrus was positively correlated with the depression score.

**Conclusion:**

Our results suggested that the ACC may be an important biomarker in MM, and its structural and functional impairments are significantly associated with the severity of pain and pain-related impairment of emotion in patients with MM. These findings demonstrated that headache-associated structural and functional abnormalities in the ACC may can provide integrative evidence on the physiological mechanisms of MM.

## Introduction

Migraine, a primary headache disorder, is classically characterized by unilateral, throbbing, and pulsating headaches associated with photophobia, phonophobia, nausea, and vomiting (GBD 2016 Headache Collaborators, 2018). Menstrual migraine (MM), a common subtype of migraine, is a migraine that occurs in women during their menstrual cycle (Bharadwaj et al., 2021; Vetvik and MacGregor, 2021). It has been reported that 42–70% of female migraineurs experience aggravated migraine attacks during the menstrual period (Vetvik et al., 2010, 2014; Bharadwaj et al., 2021). Vetvik et al. (2015) demonstrated that MM attacks were longer and more frequently associated with severe nausea than were non-menstrual migraine attacks. MM attacks frequently lead to mood disturbance (Ferreira et al., 2017; Blumenfeld et al., 2021; Zhang et al., 2021a) such as anxiety and depression. Moreover, while pharmacological treatment is recommended for migraine, patients with MM are less responsive to medication (Nierenburg et al., 2021; Vetvik and MacGregor, 2021). Therefore, MM has become a serious global health problem.

To control MM effectively, its pathophysiology must be clearly understood. Neuroimaging approaches have been adopted to investigate brain structural and functional changes in migraine (Evans et al., 2020; Ashina et al., 2021; Messina et al., 2021). Kim et al. (2008) found that repeated migraine attacks could selectively damage several brain regions (insula, prefrontal, cingulate, and posterior parietal gyrus). One study (Maleki et al., 2012) illustrated that female migraine patients had thicker posterior insula and precuneus compared with healthy controls (HCs). By using graph theory analyses, our previous study (Liu et al., 2015) revealed that resting-state functional connectivity (rsFC) was altered in patients with chronic migraine. However, the pathogenesis of the MM subtype of migraine remains unclear. Thus, it would be valuable to use neuroimaging technology to explore the pathogenic mechanisms underlying MM.

According to previous studies, MM is a multidimensional, central nervous system disease associated with structural and functional alteration of the brain (Kim et al., 2008; Maleki et al., 2012; Liu et al., 2015; Xu et al., 2020). Thus, a relationship may exist between brain variables and disease-related clinical disabilities such as the severity and frequency of pain and emotionality. Nevertheless, the underlying neuropathological mechanism remains unclear. Moreover, many neuroimaging studies of migraine have focused solely on changes in brain structure or functional architecture (Schwedt et al., 2015; Jia and Yu, 2017; Yu et al., 2021; Zhang et al., 2021b). A more vital issue that has not been researched is whether and how the observed structural alterations are associated with functional deficits in migraine (Li et al., 2020). Therefore, investigating both structural and functional changes in one sample can clarify the neural mechanisms of MM and guide the choice of treatment options.

Based on the above, we hypothesized that patients with MM may show concurrent brain structural and functional differences if investigated using integrated and advanced whole brain data-driven analytical approaches. Examples are voxel-based morphometry (VBM), to assess the alterations of gray matter volume (GMV) (Ashburner and Friston, 2000), and seed-based functional connectivity (FC), to reveal collective abnormalities in information exchange within the brain caused by disease (Smitha et al., 2017). To focus the research hypothesis, we first identified the specific brain GMV differences between the MM and healthy control (HC) groups using VBM. We then used seed-based FC analysis to assess the functional changes in the identified brain areas in the MM group. In this process, a correlation analysis was carried out to investigate the relationship between the observed VBM and rsFC alterations and the clinical variables, to confirm that these alterations contribute to MM clinicopathology.

## Materials and methods

This study was registered in the Chinese Clinical Trial Registry (registration number: ChiCTR-IOR-15006648) and was approved by the Sichuan Regional Ethics Review Committee on Traditional Chinese Medicine (TCM; ethical approval number: 2015KL-004). Participants were enrolled from two hospitals in Chengdu, Sichuan, China: The Hospital of Chengdu University of TCM, and The Chengdu Integrated TCM and Western Medicine Hospital. Recruitment was conducted between June 2015 and August 2018. This study was conducted in accordance with the Declaration of Helsinki (World Medical Association, 2013). Voluntary written informed consent was obtained from each subject after verbal and written explanation of the study.

### Participants

Seventy-five patients with MM were recruited from the Departments of Neurology and Gynecology of the participating hospitals. These patients were diagnosed according to the International Classification of Headache Disorders, 3rd edition, beta version (ICHD-3beta) Headache Classification Committee of the International Headache Society [IHS] (2018). The inclusion criteria required all subjects to be female, aged 18–50 years old, right-handed, have no more than six migraine attacks per month outside of the menstrual cycle, have a history of menstrual migraine without aura for 6 months or more, and have a stable 28 (± 7) day menstrual cycle. Patients with any of the following conditions were excluded: neurological diseases; any of hypertension, diabetes mellitus, hypercholesterolemia, vascular/heart disease, or any major systemic condition; pregnancy or lactation; history of alcohol or drug abuse; any neuroimaging research study participation during the previous 6 months; and inability to understand the investigator’s instructions.

Fifty-four female HCs were recruited from the community and were matched to the patients for age, height, and weight. The exclusion criteria were as follows: left-handedness, migraine, chronic pain, previous vestibular neuritis, Meniere’s disease, secondary somatoform vertigo, drug abuse, neurological, mental, or systemic disorders, ischemic or hemorrhagic stroke, or severe head trauma.

The patients were instructed and agreed not to take any prophylactic medication during the observation period. In cases of severe pain, ibuprofen (300 mg per capsule with sustained release) was used as rescue medication during the observation period. Patients with migraines in this study were not in their menstruation period and had been migraine-free for at least 72 h before the magnetic resonance imaging (MRI) scan. After scanning, all participants reported being awake and free of any headache or migraine.

### Clinical variables

The demographic information including age, weight, and height of the participants was recorded. The clinical characteristics of patients with MM were assessed according to the International Headache Society (HIS) Guidelines for Migraine Clinical Trials (Tassorelli et al., 2018), based on the patients’ headache diaries. The clinical outcomes were measured every 4 weeks, and headache attack frequency (the number of migraines separated by pain-free intervals of at least 48 h of headache) and headache intensity were used as outcome indicators. A 10-point visual analogue scale (VAS) from 0 (no pain) to 10 (worst pain ever) was used to rate headache intensity. In addition, the self-rating anxiety scale (SAS) and the self-rating depression scale (SDS) were used to assess negative emotions in patients with MM (Janus et al., 1988; Seidel et al., 2009; Zhang et al., 2021a).

### Magnetic resonance imaging data acquisition

Magnetic resonance imaging was performed during the periovulatory phase (days 12–16 of the menstrual cycle) (Janus et al., 1988). MRI data were acquired using a GE Discovery MR750 3.0 T system with an eight-channel, phased array head coil (General Electric, Milwaukee, WI, United States). Functional MR images were obtained using a single-shot gradient-echo echo-planar imaging sequence with the following parameters: repetition time = 2,000 ms; echo time = 25 ms; flip angle = 90°; field of view = 240 mm × 240 mm; matrix = 64 × 64; slice thickness = 3 mm; and voxel size = 3.44 mm × 3.44 mm × 4 mm. Participants were instructed to keep their eyes closed and stay awake during the entire functional scan.

### Data analysis

#### Clinical data analysis

Baseline demographic information was analyzed using SPSS (version 26.0; SPSS Inc., Chicago, IL, United States). Continuous variables are presented as means with 95% confidence intervals (CIs). Categorical variables are presented as n (percentage). Between-group comparisons were performed using two-sample *t*-tests and the two-tailed χ^2^ test as appropriate. *P*-values less than 0.05 were considered statistically significant.

#### Voxel-based morphometry analysis

In this study, the VBM8 toolbox^1^ implemented in SPM8^2^ was employed to preprocess the structural T1-weighted images. First, the images were checked for artifacts and the origins of the images were adjusted to the anterior commissure. Subsequently, the images of each participant were normalized to the standard Montreal Neurological Institute template using an affine followed by non-linear transformation and resampling to 1.5 mm × 1.5 mm × 1.5 mm. The normalized images were segmented into GMVs, white matter volumes, and cerebrospinal fluid volumes. The data quality of the segmented maps was then checked. The probabilistic GM maps were further smoothed using a 6-mm full-width-at-half-maximum (FWHM) Gaussian kernel. The smoothed GM images were analyzed as follows (Ashburner and Friston, 2000).

Two-sample *t*-tests were used to compare patients and healthy subjects by assigning the total intracranial volume as a covariate of no interest. The significance of group differences was set at *P* < 0.05 using the Gaussian random field (GRF) correction (voxel, *P* < 0.01; cluster, *P* < 0.05) (Yu et al., 2021).

Thereafter, we extracted the average values of regions of interests (ROIs) with variation in GMV and performed Pearson correlation analyses with all parameters, including headache frequency and headache intensity. Age and whole-brain volume were controlled for as covariates. The significance threshold was set at *P* < 0.05.

#### Functional connectivity analysis

The rs-fMRI data preprocessing and seed-based FC analysis were performed using the Data Processing Assistant for Resting-State fMRI DPABI V2.3 software in the MATLAB environment. The first 10 volumes were not analyzed to allow for signal equilibration effects. The fMRI images were slice-timing corrected, head-motion corrected (subjects with head motion exceeding 2 mm or 2° of rotation were excluded), the Friston 24 motion parameters, the white matter signal, and the cerebrospinal fluid (CSF) signal were regressed out, the images were normalized using structural image unified segmentation, and then re-sampled to 3-mm cubic voxels. The data were then detrended, bandpass filtered from 0.01 to 0.08 Hz, and smoothed with a 6-mm FWHM Gaussian kernel for FC analysis (Yan et al., 2016).

To calculate the differences in FC across brain regions in patients with MM, rsFC analysis was carried out using the brain regions associated with clinical indicators as seeds. The average time course was obtained from the seed, and correlation analysis was then performed in a voxel-wise manner. The correlation coefficients were converted into *z*-values using Fisher’s r-to-z transformation. Contrast images were generated for each subject by estimating the regression coefficient between all brain voxels and each seed time series. A comparison of FC between the groups was performed using a two-sample *t*-test in SPM, with age as a covariate. Multiple-comparisons correction was performed using a Gaussian random field at *P* < 0.05 (voxel, *P* < 0.01; cluster, *P* < 0.05) (Jin et al., 2013).

Finally, brain regions showing significant differences based on the results of two-sample *t*-testing were defined as ROIs for further analysis. ROIs were defined as 6-mm spheres centered at the position of peak statistical difference. we extracted the average *z*-values for ROIs with significant differences and performed Pearson correlation analysis with the patients’ clinical symptoms using SPSS 26.0. Age was controlled for as a covariate. The significance threshold was set at *P* < 0.05.

## Results

### Clinical data

We found no statistically significant differences between the MM and HC groups in terms of age, height, and weight (*P* > 0.05) (Table 1).

**TABLE 1:** Demographic and clinical characteristics of patients.

Characteristic	MM (*n* = 75)	HC (*n* = 54)	*P*
Age (year)	33.09 ± 6.94	32.67 ± 12.51	0.81
Height (cm)	159.61 ± 4.42	159.51 ± 5.58	0.91
Weight (kg)	53.11 ± 6.04	52.10 ± 4.19	0.29
Headache frequency (days/month)	3.23 ± 1.95	–	–
VAS score	4.55 ± 1.77	–	–
SAS score	47.76 ± 11.19	–	–
SDS score	47.33 ± 12.31	–	–

All entries are means with 95% confidence intervals. VAS, visual analog scale; SAS, self-rating anxiety scale; SDS, self-rating depression scale; MM, menstrual migraine; HC, healthy controls.

### Voxel-based morphometry results

**FIGURE 1:**
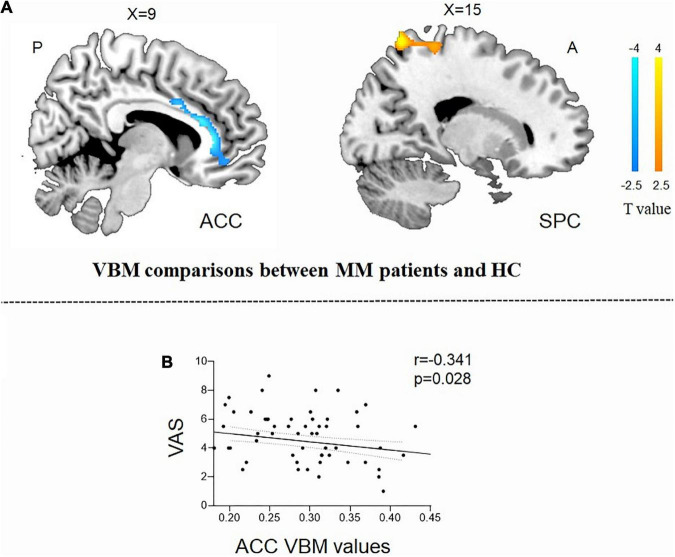
Structural analysis results of patients with menstrual migraine compared with healthy controls (*P* < 0.05, Gaussian random field-corrected). **(A)** Patients with MM showed decreased GM volumes in the ACC and increased GM volumes in the superior parietal cortex as compared to HCs. **(B)** Reduced GM volume in the ACC was correlated with headache intensity (*P*-uncorrected). ACC, anterior cingulum cortex; GM, gray matter; VAS, visual analog scale; SPC, superior parietal cortex; MM, menstrual migraine patients; HC, healthy control.

Compared with the HCs, the patients with MM showed a significantly decreased GMV in the right anterior cingulum cortex (ACC) and an increased GMV in the superior parietal cortex (SPC) (Table 2 and Figure 1A). In patients with MM, only the GMV in the right ACC showed a significant inverse correlation with the VAS score (*r* = −0.341, *P* = 0.028, uncorrected) (Figure 1B).

**TABLE 2:** Voxel-based morphometry comparisons between patients with menstrual migraine (MM) and healthy controls (HCs).

Brain region	L/R	Cluster size, voxels	MNI coordinates			*T*-value	BA
			x	y	z		
Anterior cingulum cortex	R	987	9	34	6	−4.16	11
Superior parietal cortex	R	675	15	−60	70	4.50	7

P < 0.05, Gaussian random field corrected. BA, Brodmann area; MNI, Montreal Neurological Institute; L, left; R, right.

### Functional connectivity results

**FIGURE 2:**
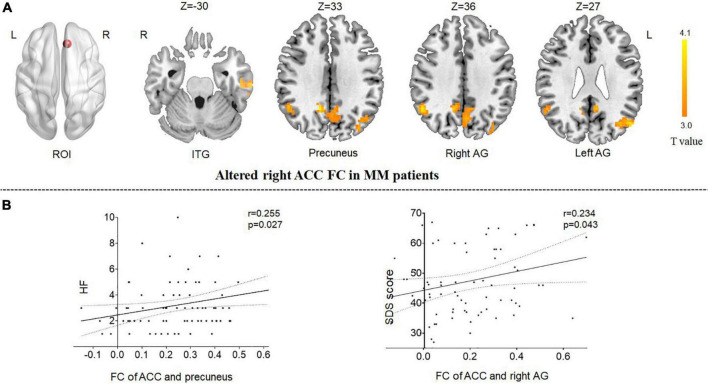
Functional analysis results of the right ACC in the resting state in patients with MM compared with HCs (*P* < 0.05, Gaussian random field-corrected). **(A)** Patients with MM showed increased FC between the right ACC and the left ITG, the right precuneus, and the bilateral AG as compared to HCs. **(B)** The FC changes in the right ACC, right precuneus, and right angular gyrus were positively correlated with clinical indicators (*P*-uncorrected). ACC, anterior cingulum cortex; AG, angular gyrus; FC, functional connectivity; HF, headache frequency; ROI, regions of interest; ITG, inferior temporal gyrus; HC, healthy control; MM, menstrual migraine patient.

Because the reduction in the GMV of the ACC was related to clinical symptoms, the ACC region was regarded as the region of interest from which to explore the correlation with other brain areas using rsFC analysis. Compared with HCs, patients with MM showed increased FC between the right ACC and the left inferior temporal gyrus (ITG), the right precuneus, and the bilateral angular gyrus (AG). Patients showed no decrease in FC between the right ACC and any other brain region compared with HCs (Table 3 and Figure 2A).

**TABLE 3:** Altered right anterior cingulum cortex functional connectivity in patients with MM.

Brain region	L/R	Cluster size, voxels	MNI coordinates			*T*-value	BA
			X	Y	Z		
Inferior temporal gyrus	L	341	−54	−12	−30	4.5254	20
Precuneus	R	577	12	−54	33	4.2327	18
Angular gyrus	R	348	51	−54	36	4.0041	39
Angular gyrus	L	361	−51	−69	27	3.8344	39

P < 0.05, Gaussian random field corrected. BA, Brodmann area; MM, menstrual migraine; MNI, Montreal Neurological Institute, L, left; R, right.

The correlation results showed that (1) the altered FC between the right ACC and the right precuneus was positively correlated with headache frequency (*r* = 0.255, *P* = 0.027, uncorrected); and (2) the altered FC between the right ACC and the right AG was positively correlated with the SDS score (*r* = 0.234, *P* = 0.043, uncorrected) (Figure 2B).

## Discussion

To the best of our knowledge, no previous neuroimaging study has combined VBM and FC analyses to determine the association between GMV and FC changes in patients with MM as well as their correlation with pain. In the present study, we used VBM and FC analysis to evaluate functional and structural changes in GMV patterns in patients with MM. Compared with HCs, patients with MM exhibited brain GM structural abnormalities in the right ACC and right SPC. In addition, we observed increases in FC between the left ACC (where GMV was relatively reduced in patients) and the right precuneus, the left ITG, and the bilateral AG. These findings suggest that abnormalities in the default-mode network (DMN) associated with pain may be involved in the pathophysiology of MM. Abnormalities in these brain regions associated with subjective pain and negative emotions, which supports our hypothesis. Our results may provide specific and more reliable insights into ongoing pain changes in patients with MM and can form a basis for elucidating the physiological and pathological mechanisms underlying MM.

### Changes in the gray matter volume of the anterior cingulum cortex and superior parietal cortex in patients with menstrual migraine

Previous studies have shown that patients with migraine have reduced GMV in the ACC relative to HCs (Jin et al., 2013; Russo et al., 2020; Yu et al., 2021). Because MM is a subtype of migraine, the main findings of this study are consistent with those of previous studies on migraine. The ACC is a limbic brain structure that is consistently activated across pain modalities, as supported by lesion and imaging studies (Schnitzler and Ploner, 2000; Duerden and Albanese, 2013). Pain can cause ACC activation: a neuroimaging meta-analysis suggested that ACC activation is central to pain perception and is a core region involved in pain processing (Jensen et al., 2016). Overall, the perigenual ACC was found to be less activated on fMRI in patients with chronic pain than in HCs (Noll-Hussong et al., 2013) and is thought to play a pivotal role in the central pain control system (Garcia-Larrea and Peyron, 2007). The ACC of patients with migraine, irrespective of the migraine type, has decreased GMV, which is worsened by repeated headaches (Yu et al., 2021). In patients with MM, the value of bilateral ACC voxel-mirrored homotopic connectivity was significantly increased, suggesting that spontaneous ACC brain function was enhanced (Zhang et al., 2018). It was speculated that this might be related to the influence of increasing the pain threshold on the medial pain pathway. Li et al. (2021) suggested that menstrual-related migraines are associated with cerebral hyperconnectivity and hyperperfusion in critical pain-processing brain regions. VBM studies have identified an increase in GMV in the SPC in patients with MM, and the SPC of migraine patients was activated when accompanied by painful stimulation (Zhang et al., 2017). The parietal cortex plays a key role in human pain avoidance, particularly in migraine patients (Gandhi et al., 2022). Thus, the ACC and SPC mediate structural changes associated with migraine, including MM, while demonstrating that disruption of the ACC and SPC structures causes abnormal modulation of pain perception in patients with MM.

### Abnormalities of the anterior cingulum cortex–default-mode network in patients with menstrual migraine

Because the reduction in the GMV of the ACC is related to clinical symptoms, the ACC region was regarded as the region of interest for exploring connectivity with other brain areas using rsFC analysis. This revealed that, in comparison with HCs, the rACC region had increased FC with the ITG, the precuneus, and the AG in migraineurs. In our previous studies (Liu et al., 2012; Yuan et al., 2013), longer disease duration was correlated with increased FC between the ACC and other regions. Other studies (Tan et al., 2017; Juarez-Salinas et al., 2019) have revealed that inhibition of ACC function could effectively relieve neuropathic pain. Moreover, the ACC is engaged in a response to unpleasant emotional experiences related to pain (Peyron et al., 2000; Ellerbrock et al., 2013). Thus, an abnormal functional network in which the ACC is the seed region of interest is closely related to pain and negative emotions.

The ITG is a key structure of the brain for the development and maintenance of pain. Obermann et al. (2014) found that the GMV of the ITG was decreased in migraineurs (Wei et al., 2021). Moreover, Wei et al. (2021) reported an increase in FC with the ITG and other brain regions in migraineurs without aura. Our findings confirmed these results in MM. Moreover, Lee et al. (2017) demonstrated that the ITG region is correlated with a complex perception of menstrual pain (Wang et al., 2020). Thus, the ACC–ITG network may be related to pain perception.

The precuneus has been shown to be associated with pain sensitivity (Emerson et al., 2014; Goffaux et al., 2014; Qin et al., 2020; Rolls et al., 2020a,b). Goffaux et al. (2014) demonstrated that pain-induced responses in the precuneus are closely related to pain sensitivity in HCs. Emerson et al. (2014) discovered a negative correlation between GM density in the precuneus and pain sensitivity. Moreover, Pan et al. (2020) found that pain sensitivity varies depending on headache frequency. In our previous study, based on dynamic FC, precuneus was found to be significantly associated with headache frequency in migraineurs (Tu et al., 2019). These previous reports are consistent with the present finding that the functional network between the ACC and the precuneus is associated with headache frequency. Thus, alterations in the connectivity between the ACC and precuneus may result in enhanced pain sensitivity, which could contribute to MM pathology.

The functional network between the ACC and AG was positively correlated with negative emotions in patients with MM. Many neuroimaging studies (Duggirala et al., 2020; Rolls et al., 2020a,b) have proven that emotional disorders are related to abnormal changes in the AG. Moreover, sex is known to be one of the important factors influencing migraine attacks. Multiple studies (Maleki et al., 2012; Zhao et al., 2017; Tsai et al., 2022) have proven a substantial differential effect of the disease on female as compared to male migraineurs, such as higher scores for negative emotions in female migraineurs. Our fMRI study of patients with MM was consistent with the above findings. Our identification of an abnormal ACC–AG FC may advance the understanding of the mechanism of pain associated with negative emotions in patients with MM. These findings may also help explain the relationship between MM pain and future mental illness.

Furthermore, the DMN is a robust and well-studied brain network, comprising the ACC, ITG, AG, precuneus, and other regions (Jacobs et al., 2016; Cunningham et al., 2017; Deng et al., 2019; Jones et al., 2020). Numerous studies in chronic pain patients have demonstrated abnormal functional connectivity between the DMN and the pain network (Farmer et al., 2012). The ACC, ITG, AG, and precuneus are considered core brain regions that may be closely related to MM, suggesting that disruption of the DMN may lead to abnormal brain regulation in patients with MM. Thus, the DMN may play an important role in MM.

### Abnormal brain regions correlation with clinical symptoms in patients with menstrual migraine

To better explore the possible physiological mechanisms underlying the structural and functional changes in patients with MM, we also performed a correlation analysis. The main results were that the GMV of the ACC increased with increasing headache intensity, the FC between the ACC and the precuneus increased with increasing headache frequency, and the FC between the ACC and the AG was influenced by emotionality accompanied by headache. Pain can cause ACC activation (Jensen et al., 2016). Abnormalities in the precuneus are commonly reported to correlate with migraine frequency (Tu et al., 2019). In addition, the AG is regarded as a key brain area in emotional disorders (Duggirala et al., 2020; Rolls et al., 2020a,b). The current findings suggest that these regions are positively associated with the severity of the pain and negative emotions of MM. Furthermore, our findings suggest that the DMN may contribute to the severity of headaches in patients with MM.

### Limitations

This study had several limitations. First, the sample size of the included patients with MM differed from that of the HCs, which may have limited the recognition of additional differences. Second, in the correlation analysis, the number of different regions and clinical indicators was large, with the result that the correlations were relatively weak and not corrected for multiple comparisons; therefore, our structural analysis was limited. In addition, the current study could not establish whether the reduction in the ACC GMV was caused by pain or by the disease itself. Moreover, in a descriptive study such as this, we cannot identify direct cause-and-effect associations. Furthermore, longitudinal fMRI studies are needed to establish and verify the findings.

## Conclusion

Our integrated structural and functional approach explored brain abnormalities in MM. An abnormal structure of the ACC was found in patients with MM, and an abnormality of the DMN was found with the ACC as the seed region. These abnormalities were associated with clinical manifestations, illustrating the influence of the ACC on the neural mechanisms of pain in MM. These findings provide new insights into the pathophysiological mechanisms of MM through structural and functional evidence.

## Data availability statement

The original contributions presented in this study are included in the article/supplementary material, further inquiries can be directed to the corresponding authors.

## Ethics statement

The studies involving human participants were reviewed and approved by Sichuan Regional Ethics Review Committee on Traditional Chinese Medicine. The patients/participants provided their written informed consent to participate in this study. Written informed consent was obtained from the individual(s) for the publication of any potentially identifiable images or data included in this article.

## Author contributions

Z-WW and LZ conceived and designed the study. Z-WW, Y-TZ, TX, J-RD, YW, YZ, and H-QL recruited the participants. Z-WW and XW analyzed the data. Z-WW and Z-HY drafted the manuscript. Z-WW, Z-HY, XW, F-RL, and LZ revised the manuscript. All authors contributed to the article and approved the submitted version.

## Footnotes

1
http://dbm.neuro.uni-jena.de/vbm8

2
http://www.fil.ion.ucl.ac.uk/spm
